# Heterologous Expression and Biochemical Characterization of the Human Zinc Transporter 1 (ZnT1) and Its Soluble C-Terminal Domain

**DOI:** 10.3389/fchem.2021.667803

**Published:** 2021-04-30

**Authors:** Camila A. Cotrim, Russell J. Jarrott, Andrew E. Whitten, Hassanul G. Choudhury, David Drew, Jennifer L. Martin

**Affiliations:** ^1^Griffith Institute for Drug Discovery, Griffith University, Nathan, QLD, Australia; ^2^Australian Nuclear Science and Technology Organisation, Lucas Heights, NSW, Australia; ^3^Institute for Molecular Bioscience, University of Queensland, St Lucia, QLD, Australia; ^4^Department of Biochemistry and Biophysics, Stockholm University, Stockholm, Sweden; ^5^Vice-Chancellor's Unit, University of Wollongong, Wollongong, NSW, Australia

**Keywords:** human zinc transporter 1, cation diffusion facilitator, C-terminal domain, small-angle X-ray scattering, membrane proteins

## Abstract

Human zinc transporter 1 (hZnT1) belongs to the cation diffusion facilitator (CDF) family. It plays a major role in transporting zinc (Zn^2+^) from the cytoplasm across the plasma membrane and into the extracellular space thereby protecting cells from Zn^2+^ toxicity. Through homology with other CDF family members, ZnT1 is predicted to contain a transmembrane region and a soluble C-terminal domain though little is known about its biochemistry. Here, we demonstrate that human ZnT1 and a variant can be produced by heterologous expression in *Saccharomyces cerevisiae* cells and purified in the presence of detergent and cholesteryl hemisuccinate. We show that the purified hZnT1 variant has Zn^2+^/H^+^ antiporter activity. Furthermore, we expressed, purified and characterized the soluble C-terminal domain of hZnT1 (hZnT1-CTD) in a bacterial expression system. We found that the hZnT1-CTD melting temperature increases at acidic pH, thus, we used an acetate buffer at pH 4.5 for purifications and concentration of the protein up to 12 mg/mL. Small-angle X-ray scattering analysis of hZnT1-CTD is consistent with the formation of a dimer in solution with a V-shaped core.

## Introduction

Zinc (Zn^2+^) plays an important role in many key biological processes, such as immune function, redox signaling, and cell death (Vallee and Falchuk, 1993). Conversely, Zn^2+^ dysregulation can lead to chronic inflammation, growth retardation or metabolic disorders (Prasad, 2013). Moreover, Zn^2+^ has been associated with other medical conditions, including diabetes, Alzheimer's disease and transient neonatal zinc deficiency (TNZD) (Lovell et al., 2005; Chowanadisai et al., 2006; Sladek et al., 2007). High levels of Zn^2+^ are toxic and tight regulation of intracellular Zn^2+^ concentrations is essential to maintain good health (Huang and Tepaamorndech, 2013). In mammals, physiological Zn^2+^ levels are regulated by the action of three classes of proteins: metallothioneins (MTs); ZRT/IRT-like protein (ZIPs) and cation diffusion facilitators (CDF) (Blindauer, 2015; Kolaj-Robin et al., 2015). MTs bind free Zn^2+^ ions directly (Kimura and Kambe, 2016), whereas CDF and ZIP proteins regulate intracellular Zn^2+^ levels by sequestration and recruitment from organelles or the extracellular environment (Huang and Tepaamorndech, 2013).

In mammals, the zinc transporter CDF family members are referred to as ZnT or solute carrier family 30 (SLC30A). To date, ten members of the ZnT family (ZnT1 - ZnT10) have been identified (Huang and Tepaamorndech, 2013). Predicted structures of eukaryotic ZnT transporters have been reported on the basis of the crystal structure and cryo-EM structures of *Escherichia coli* YiiP (EcYiiP) (Lu and Fu, 2007) and *Shewanella oneidensis* YiiP (SoYiiP) (Coudray et al., 2013; Lopez-Redondo et al., 2018). Recently, cryo-EM structures of human ZnT8 (hZnT8) in different conformations were reported (Xue et al., 2020) revealing its overall structure and shedding light on its mechanism of action. As observed for the bacterial zinc transporter YiiP structures, hZnT8 forms a Y-shaped homodimer with two domains: a transmembrane domain (TMD) comprising six transmembrane helices, and a cytosolic C-terminal domain (CTD) (Lu and Fu, 2007; Lu et al., 2009; Coudray et al., 2013; Lopez-Redondo et al., 2018; Xue et al., 2020). Several CDF proteins have been described as antiporters, catalyzing active efflux of metals (M^2+^) by a proton-motive force in exchange for H^+^ or K^+^ (Guffanti et al., 2002; Chao and Fu, 2004; Ohana et al., 2009; Shusterman et al., 2014). The transport mechanism of individual CDF members has included divergent mechanisms, such as scissoring and alternating-access models (see Cotrim et al., 2019, for review). The recent hZnT8 structures in the outward- and inward-facing conformations suggest a simple two-state model of Zn^2+^ transport, as no occluded state has been reported in the structural analysis (Xue et al., 2020).

Among ZnT members, ZnT1 is the most ubiquitously expressed and the only member localized to the plasma membrane (Palmiter and Findley, 1995; Qin et al., 2009), where it plays a pivotal role in zinc homeostasis by conferring resistance against zinc toxicity (Palmiter and Findley, 1995). Previous studies have suggested that ZnT1 is essential to embryonic development, as knockout of the *SLC30A1* gene in mice is lethal during early embryogenesis (Langmade et al., 2000; Andrews et al., 2004). Furthermore, altered expression levels of *SLC30A1* have been linked to Alzheimer's disease and different types of cancers (Lovell et al., 2005; Jing et al., 2018). A recent study also suggested that altered expression of ZnT1 may contribute to pro-oncogenic processes and cancer progression (Lehvy et al., 2019). In addition to its function as a zinc transporter, ZnT1 acts as a negative regulator of the L-type calcium channel (LTCC) through its interaction with the β_2a_-subunit of voltage-gated calcium channels (Segal et al., 2004; Levy et al., 2009). Recent studies have also demonstrated that the soluble C-terminal domain (CTD) of ZnT1 interacts with Raf-1 kinase leading to activation of the Ras-Raf-ERK signaling pathway, which in turn may promote a cardioprotective effect from ischemia-reperfusion (Jirakulaporn and Muslin, 2004; Beharier et al., 2012).

Although the cellular importance of ZnT1 has been widely investigated, little is known about its biochemical features. In this study, we investigated the production of human ZnT1 (hZnT1) recombinantly expressed in *Saccharomyces cerevisiae*. Our results indicate that full-length and a truncated form of hZnT1 can be produced in yeast and that protein extraction in the presence of detergent incorporated with cholesteryl hemisuccinate generates active protein. We also expressed, purified and investigated the soluble CTD of human ZnT1 (hZnT1-CTD) by small-angle X-ray scattering (SAXS), showing that it forms dimers in solution. Taken together, our results provide the basis for further structural investigations of ZnT proteins.

## Materials and Methods

### Protein Expression

#### Protein Expression in Yeast Cells

**Figure 1:**
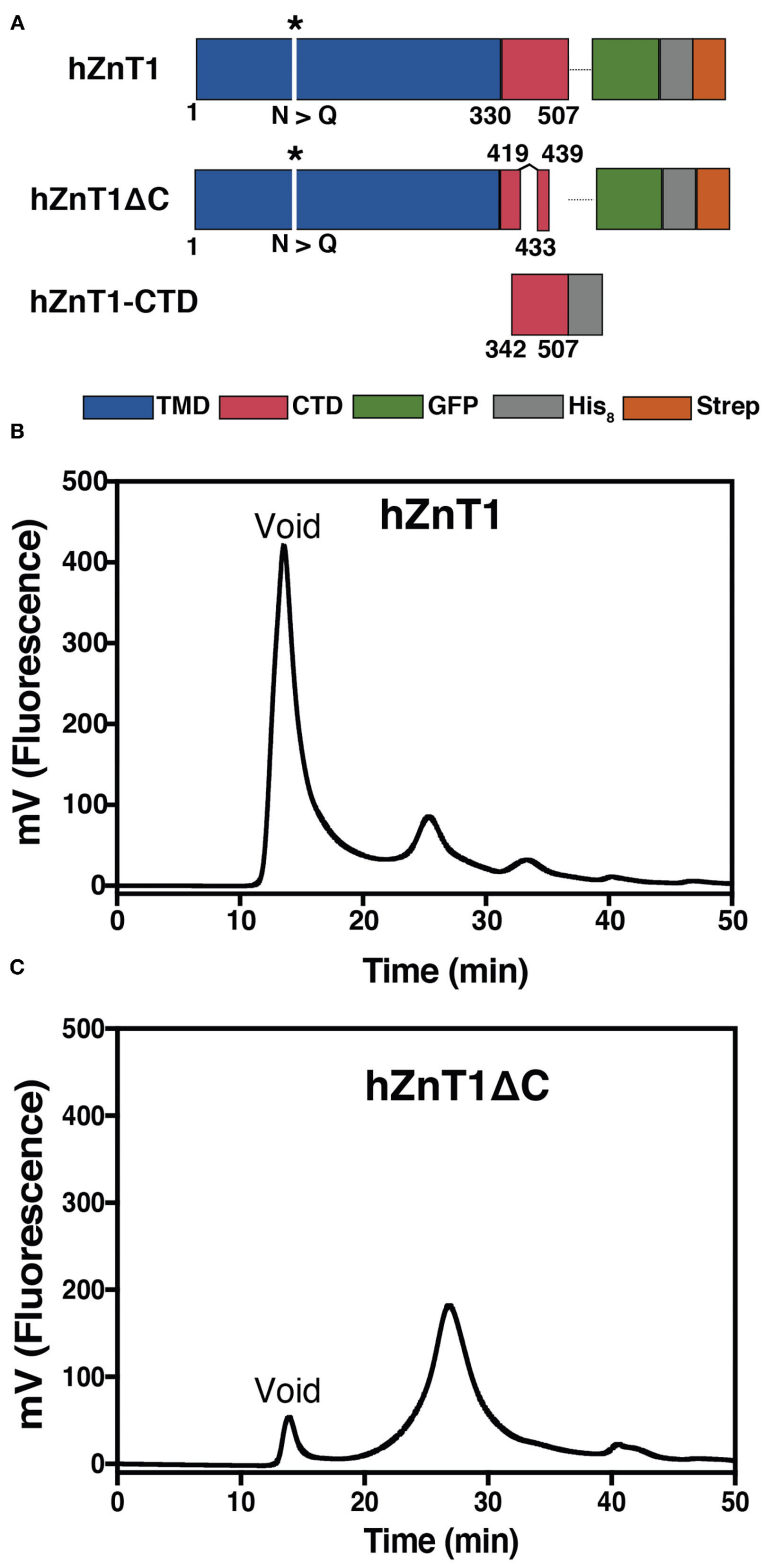
Production of hZnT1 and hZnT1ΔC. **(A)** Linear representation of the constructs. Transmembrane domain is colored in blue, the soluble C-terminal domain (CTD) in red, with the white spaces indicating regions that were truncated. GFP is shown in green and the His_8_ and Strep tags are shown in gray and orange, respectively. The asterisk indicates mutation of N99Q. **(B)** Fluorescence size exclusion chromatography (FSEC) profile of purified hZnT1. **(C)** hZnT1ΔC optimized FSEC profile. Profiles based on GFP fluorescence. Wavelengths: 488 nm (excitation); 512 nm (emission). Column: Superose 6; flow: 0.5 mL/min; buffer: 20 mM Tris-HCl pH 7.6, 150 mM NaCl, 0.03% DDM.

We used a variant of human ZnT1 in which Asn99 (potential glycosylation site) was replaced with Gln. We worked with two constructs, both codon-optimized for *S. cerevisiae* expression: hZnT1 [see Supplementary Note 1 in Supplementary Material for sequence (GenScript)] and hZnT1ΔC. The latter construct was designed to remove C-terminal regions between Glu420-Pro432 and Gln440-Leu507 that were predicted to be highly disordered. Both constructs were inserted into a modified pDDGFP-2 vector (Newstead et al., 2007) (encoding C-terminal His- and Strep-tags) by homologous recombination in *S. cerevisiae* strain FGY217 (MATα, ura3-52, lys2Δ201 and pep4Δ) (Kota et al., 2007; Drew et al., 2008) (Figure 1A) (see Supplementary Table 1 for list of primers). hZnT1 and hZnT1ΔC were expressed in *S. cerevisiae* FGY217 in -URA selective medium containing 0.1% (w/v) glucose (Drew et al., 2008). Cells were grown at 30°C till OD_600_ 0.6 and protein expression was induced by 2% (w/v) galactose for 22 h at 30°C. After expression, cells were harvested by centrifugation (Sorvall SuperLynx, Thermo Fisher Scientific), resuspended in CRB buffer (50 mM Tris-HCl pH 7.6, 1 mM EDTA, 0.6 M sorbitol), frozen and stored at −80°C.

For membrane fraction preparation, cells were lysed by mechanical disruption and unbroken cells and debris were removed by centrifugation at 10,000 g at 4°C for 10 min. Membranes were then isolated at 4°C by ultracentrifugation (Sorvall WX+ ultracentrifuge, Thermo Fisher Scientific) at 125,000 g for 2 h (Drew et al., 2008). The membrane was resuspended in 20 mM Tris-HCl pH 7.5, 0.3 M sucrose, 0.1 mM CaCl_2_ in a ratio of 3.5 mL buffer per liter of expression and stored at −80°C.

#### Protein Production in Bacteria Cells

For cloning of the C-terminal soluble domain (hZnT1-CTD), the codon-optimized gene sequence (GenScript) encoding residues 342-507 was amplified and cloned into a pET24a vector (Novagen) containing a C-terminal His_8_-tag (Figure 1A). hZnT1-CTD was expressed in Rosetta-gami (DE3) cells at 30°C for 22–24 h using autoinduction (Studier, 2005) supplemented with 20 μM ZnCl_2_. After expression, cells were harvested by centrifugation (Sorvall SuperLynx, Thermo Fisher Scientific) and the pellet stored at −80°C.

### Detergent Screening and FSEC Analysis

All detergents were obtained from Anatrace, unless otherwise stated. hZnT1 membrane was examined to measure the extraction efficiency of the following detergents: 6-cyclohexyl-1-hexyl-β-D-maltoside (Cymal-6), n-dodecyl-β-D-maltopyranoside (DDM), decyl-β-D-maltopyranoside (DM), lauryldimethylamine oxide (LDAO), neopentyl glycol (NG), n-nonyl-β-D-maltopyranoside (NM), n-octyl-β-D-glucopyranoside (β-OG), decyl glucose neopentyl glycol (DGNG) and lauryl maltose neopentyl glycol (LMNG). A stock solution of each detergent (10% (w/v)) was mixed with 500 μL membrane to a final concentration of 1% (w/v) and incubated under mild agitation for 1 h at 4°C. Samples were centrifuged (Optima^TM^ Max-XP ultracentrifuge, Beckman Coulter) at 112,000 g for 15 min to remove insolubilized material. The supernatant (containing solubilized protein) was analyzed by fluorescence size exclusion chromatography (FSEC) (Kawate and Gouaux, 2006). FSEC was carried out using an ÄKTA Purifier system (GE Healthcare) with a fluorescence detector attached (Jasco FP 2020 Plus). Samples were injected into a Superose 6 10/300 column pre-equilibrated with FSEC buffer (20 mM Tris pH 7.6, 150 mM NaCl and 0.03% DDM).

### GFP-Based Thermal Shift Assay for hZnT1 and hZnT1ΔC Proteins

Melting curves for hZnT1 and hZnT1ΔC were determined according to Nji et al. (2018). Membranes were solubilized in buffer containing 20 mM Tris-HCl pH 7.6, 150 mM NaCl, and 1% (w/v) DDM or LMNG in the presence or absence of cholesteryl hemisuccinate (CHS) for 1h at 4°C under mild agitation. After solubilization, 10% (w/v) β-OG was added to the solubilized membrane [to a final concentration of 1% (w/v)] and 150 μL of the solution transferred into 1.5 mL tubes for 10 min incubation at 4, 20, 30, 40, 50, 60, 70, 80, and 100°C using a Thermomixer (Eppendorf). Samples were centrifuged at 14,000 g (Microfuge 22R, Beckman Coulter) for 30 min at 4°C and supernatant transferred into a black Nunc 96-well optical bottom plate for measurement of GFP fluorescence. The apparent T_*m*_ was calculated by plotting the average GFP fluorescence intensity (RFU) and fitting the curves to a sigmoidal dose-response equation by GraphPad Prism software. Three technical repeats were used, with goodness of the fit >0.98.

### Protein Purification

Isolated membranes from large scale expression (36 L) of *S. cerevisiae* culture harboring overexpressed GFP-fusion proteins (hZnT1 and hZnT1ΔC) were solubilized in buffer containing PBS, 150 mM NaCl, 10% (v/v) glycerol and 2%:0.4% (w/v) DDM:CHS for 90 min at 4°C with gentle agitation. After solubilization the samples were clarified by ultracentrifugation (100,000 g, 60 min, 4°C) to remove unsolubilized material. The supernatant was incubated with 5 mL Strep-Tactin Sepharose resin (50% suspension, IBA life sciences) for 2 h at 4°C under mild agitation. The resin was transferred to a 30 mL Econo-column (BioRad) and washed with 20 column volumes (cv) of buffer containing PBS, 500 mM NaCl, 10% (v/v) glycerol; 0.03%:0.006% (w/v) DDM:CHS. The protein was eluted with buffer containing 100 mM Tris-HCl pH 8.0, 150 mM NaCl, 0.03%:0.006% (w/v) DDM:CHS, 1 mM EDTA, 50 mM biotin and elution fractions were monitored based on GFP fluorescence. Pooled fractions were further purified by size-exclusion chromatography using a Superdex 200 16/600 column pre-equilibrated with 20 mM Tris-HCl pH 7.6, 150 mM NaCl, 0.01%:0.002% (w/v) DDM:CHS connected to an ÄKTA Purifier system (GE Healthcare). Proteins were concentrated using Amicon Ultra centrifugal filter devices with a 100-kDa cutoff (Merck Millipore). Protein purity was analyzed by SDS-PAGE with Coomassie blue stain.

Isolated pellet from 2 L of Rosetta-gami (DE3) culture harboring hZnT1-CTD was resuspended in 600 mL lysis buffer (25 mM Tris-HCl pH 7.6, 300 mM NaCl, 10% (v/v) glycerol, 10 mM imidazole, 1% (v/v) Triton X-100, 3.3 mg DNase, 0.4 mg lysozyme) and sonicated for 2 min, 100% duty cycle at 20–40 Hz for 3 cycles (Branson Sonicator) at 4°C. Lysate was clarified by centrifugation (28,000 g, 30 min, 4°C) and supernatant was incubated with 3 g PrepEase metal affinity resin (USB Corporation) for 2 h under agitation at 4°C. Supernatant-resin suspension was poured through Econo-columns (BioRad) and washed with 200 mL wash buffer 1 [25 mM Tris-HCl pH 7.6, 300 mM NaCl, 10% (v/v) glycerol, 20 mM imidazole, 1 mM Tris (2-carboxyethyl)phosphine hydrochloride (TCEP)] and 200 mL wash buffer 2 (wash buffer 1 containing 40 mM imidazole). Protein was eluted in 25 mM Tris-HCl pH 7.6, 300 mM NaCl, 10% (v/v) glycerol, 500 mM imidazole, 1 mM TCEP and further purified by size exclusion chromatography (SEC) in a Superdex 200 16/600 column (GE Healthcare) equilibrated with buffer A (25 mM CH_3_COONa pH 4.5, 100 mM NaCl, 0.5 mM TCEP, 20 μM ZnCl_2_). The final purification step was carried out in a Mono Q column (GE Healthcare) with a salt gradient of 0% to 100% buffer B (buffer A containing 1 M NaCl). Fractions containing hZnT1-CTD (flow through) were pooled and concentrated as required for subsequent assays.

### Lipid Screening of Purified hZnT1ΔC

Lipid screening for purified hZnT1ΔC was carried out according to Nji et al. (2018). Briefly, to 96 μL of purified protein (0.1 to 0.3 mg/mL), 12 μL of the following lipids were added to a final concentration of 3 mg/mL: DOPC (Avanti), DOPG (Avanti), DOPE (Avanti), monoolein (Anatrace), cholesteryl hemisuccinate (CHS) (Sigma), brain lipids (Sigma), sphingomyelin (Avanti), bovine lipids (Avanti), *E. coli* total lipids (Avanti). Stock solutions of lipids were prepared to a final concentration of 30 mg mL^−1^ by solubilization in 10% (w/v) DDM overnight at 4°C. β-OG (10% (w/v) stock) was also added to the mixture to a final concentration of 1% (w/v). The samples were heated for 10 min at a temperature 5°C higher than the apparent T_*m*_ and then centrifuged at 14,000 g at 4°C (Microfuge 22R, Beckman Coulter). The supernatant was collected and GFP fluorescence was measured as previously described.

### Crystallization of hZnT1 Variants

Crystallization screening of hZnT1 variants was performed at the UQ ROCX facility at the University of Queensland (https://cmm.center.uq.edu.au/uq-rocx) using the hanging drop vapor diffusion technique. Crystallization plates for hZnT1 and hZnT1ΔC were set up with drops of 200 nL protein and 200 nL reservoir solution using a Mosquito robot (TTP Labtech) in 96-well plates. The following commercial kits designed for membrane proteins were used: MemGold1, MemGold2, MemStart/MemSys and MemMeso. The plates were incubated at 22°C. Protein concentrations varied from 5 to 15 mg/mL. Crystals were harvested from the plates and cryo-protected with 20% (v/v) ethylene glycol before flash frozen in liquid nitrogen. Crystal diffraction was assessed on beamline MX2 at the Australian Synchrotron.

hZnT1-CTD crystallization screening was carried out using the hanging drop vapor diffusion technique at 22°C. The following commercial kits were used: Hampton Research Index HT, Molecular Dimensions JCSG+, ShotgunEco, Molecular Dimensions PACT+, Molecular Dimensions ProPlex and an inhouse Combination screen (pH and concentration gradient using sodium malonate, ammonium sulfate, sodium chloride, lithium chloride/PEG 6000 plus buffers as required). Protein concentrations varied from 3 to 12 mg/mL.

### Protein Thermostability Assay

A master mix plate consisting of 42 buffers, salts, metals and additives with 6 controls was established (Supplementary Table 2). Purified hZnT1-CTD (1.0 mg/mL) was incubated with 8-fold Protein Thermal Shift Dye (Applied Biosystems, Life Technologies) for 30 min at 4°C. Using a 384 well plate (MicroAmp Optical, Applied Biosystems), 10 μL of each condition was aliquoted into every second well and 10 μL of dye:protein was added and mixed. Samples were analyzed in a QuantStudio 6 Flex (Applied Biosystems) and the instrument software was set to increase the temperature from 25 to 99°C with a heating rate of 0.05°C/s. Fluorescence intensity was measured with Excitation/Emission: 580/623 nm.

Protein unfolding profiles were analyzed using the Protein Thermal Shift software v1.3 (Applied Biosystems), and the peak in the derivative of the fluorescence signal as a function of temperature, the “melt” temperature (T_*m*_), provided a relative measure of protein stability.

### Coupled Proton Transport Assay Using hZnT1ΔC and *E. coli* ATP Synthase

Liposome preparation and protein reconstitution were carried out as previously described (Uzdavinys et al., 2017). Briefly, L-α-phosphatidylcholine lipids from soybean (type II, Sigma) and brain lipids (type I, Sigma) (ratio 1:1) were mixed in buffer containing 10 mM MOPS pH 6.5, 5 mM MgCl_2_, 100 mM KCl to a final concentration of 10 mg/mL and vortexed until homogenized. Lipids were flash-frozen in liquid nitrogen and thawed in a total of eight cycles before extrusion using polycarbonate filters (Whatman) with a pore size of 200 nm. For reconstitution, 250 μL of liposomes were destabilized by addition of sodium cholate [0.65% (w/v) final concentration] and mixed with 100 μg of both hZnT1ΔC and F_0_F_1_ ATP synthase from *E. coli* and incubated for 30 min at room temperature. Detergent was removed using a PD-10 desalting column (GE Healthcare) and the sample collected in 2.3 mL. For the assay, 100 μL of the proteoliposomes containing hZnT1ΔC and ATP synthase were diluted into 1.5 mL of working buffer (MOPS buffer, pH 6.5) containing 2.5 nM ACMA (9-amino-6-chloro-2-methoxyacridine, Thermo Fisher Scientific) and 130 nM valinomycin (Sigma). Proton influx was established by the addition of 130 μM ATP, as detected by a change in ACMA fluorescence (488 nm excitation; 410 nm emission). After 1 min equilibration, the activity of hZnT1ΔC was assessed by addition of 10 mM ZnCl_2_. The reaction was stopped with addition of NH_4_Cl (20 mM final concentration).

### Small-Angle X-Ray Scattering (SAXS) Analysis

SAXS data for hZnT1-CTD was collected on the SAXS-WAXS beamline at the Australian Synchrotron using an in-line SEC-SAXS sheath flow set-up (Supplementary Table 3) (Kirby et al., 2013, 2016). Data reduction was carried out using Scatterbrain software (v 2.71) (Software for acquiring, processing and viewing SAXS/WAXS data at the Australian Synchrotron), and corrected for solvent scattering and sample transmission. Contrast and partial specific volumes were determined from the protein sequences (Whitten et al., 2008), while the molecular mass was estimated from the Porod volume (Fischer et al., 2010). Data processing and Guinier analysis was performed using Primus (Konarev et al., 2003) (v 3.2). The pair-distance distribution function [*p*(*r*)] was generated from the experimental data using *GNOM* (v 4.6) (Svergun, 1992), from which *I*(0), *R*_g_ and *D*_max_ were determined. The program *DAMMIN* (v 5.3) (Svergun, 1999) was used to generate 16 dummy-atom models for each protein, assuming a prolate geometry with long axis perpendicular to the *C*_2_ symmetry axis. Of the 16 dummy-atom models generated, 15 were averaged using the program *DAMAVER* (v 2.8.0) (Volkov and Svergun, 2003) preserving the *C*_2_ symmetry, and the resolution of the averaged structure estimated using SASRES (Tuukkanen et al., 2016). The program CORAL (v 1.1) was used to generate 16 rigid-body models assuming a dimeric structure with *C*_2_ symmetry (Petoukhov et al., 2012). For the rigid-body modeling, the initial structure of hZnT1-CTD (residues 342–507) was generated using iTasser (Yang et al., 2014), where the N-terminal portion (342–422) is found to be similar to other homologous proteins (such as PDB ID: 3BYR), but the C-terminal portion (432–498) was predicted to be predominantly coil as there are no templates with a similar sequence that can be used to predict the structure. The two subunits were modeled with a nine residue flexible linker, and a nine residue flexible region was included at the C-terminus. A distance restraint of 10 Å between T53 in one protein of the complex and Y55 was included to force dimerization through the N-terminal domain in a similar manner to that observed in homologous structures. The chosen rigid-body showed the best overall fit (χ^2^ = 1.27). Data has been deposited in the SASBDB with accession ID: SASDJ85.

## Results and Discussion

### hZnT1 and hZnT1ΔC Can Be Heterologously Expressed in *S. cerevisiae*

Initial expression screening of hZnT1 showed strong GFP fluorescence indicative of good expression levels. However, fluorescence size exclusion chromatography (FSEC) analysis of the protein after DDM solubilization suggested protein aggregation (high peak at the void volume of the column, Figure 1B). Detergent screening was performed to identify whether other detergents might preserve ZnT1 integrity. However, all the detergents tested had a large aggregate peak at void volume (~8 mL) and a much smaller fluorescence peak (presumably corresponding to hZnT1) at ~11–13 mL (Supplementary Figure 1). Because there was little difference in the profiles, we chose to proceed with two mild detergents (Stetsenko and Guskov, 2017), DDM and LMNG, for large scale purifications.

Disordered regions are flexible parts of a protein that can inhibit crystallization (Deller et al., 2016). Sequence analysis using the Protein Disorder Prediction System (PrDOS) server (Ishida and Kinoshita, 2007) identified several regions predicted to be disordered in hZnT1. These include the cytosolic His-rich domain, from residues Gly139 to Asn244, and regions in the C-terminal domain (Glu419 to Leu507, Supplementary Figure 2A). The hZnT1ΔC variant, removes disordered regions in the CTD from Glu420 to Pro432 and from Gln440 to Leu507 (Figure 1A). The His-rich region in the TMD was not truncated, as it has been associated with zinc binding (Kawachi et al., 2008; Podar et al., 2012) and modulation of zinc transport activity (Fukue et al., 2018). Expression of hZnT1ΔC in yeast yielded an improved FSEC profile after solubilization in DDM, with a much reduced void volume peak (Figure 1C). Several attempts were made to remove the GFP tag for protein characterization, but the yield after this step was very low. Characterization of both hZnT1 and hZnT1ΔC proteins was therefore carried out in the presence of the GFP fusion tag. Using this approach, purification of either hZnT1 or hZnT1ΔC from a 36 L pellet using a two-step method (Strep-Tactin Sepharose resin followed by SEC) was reproducible and yielded ~1.5 mg and 2 mg of protein, respectively (Supplementary Figure 3).

### hZnT1 Can Be Stabilized by CHS and Lipids

**Figure 2:**
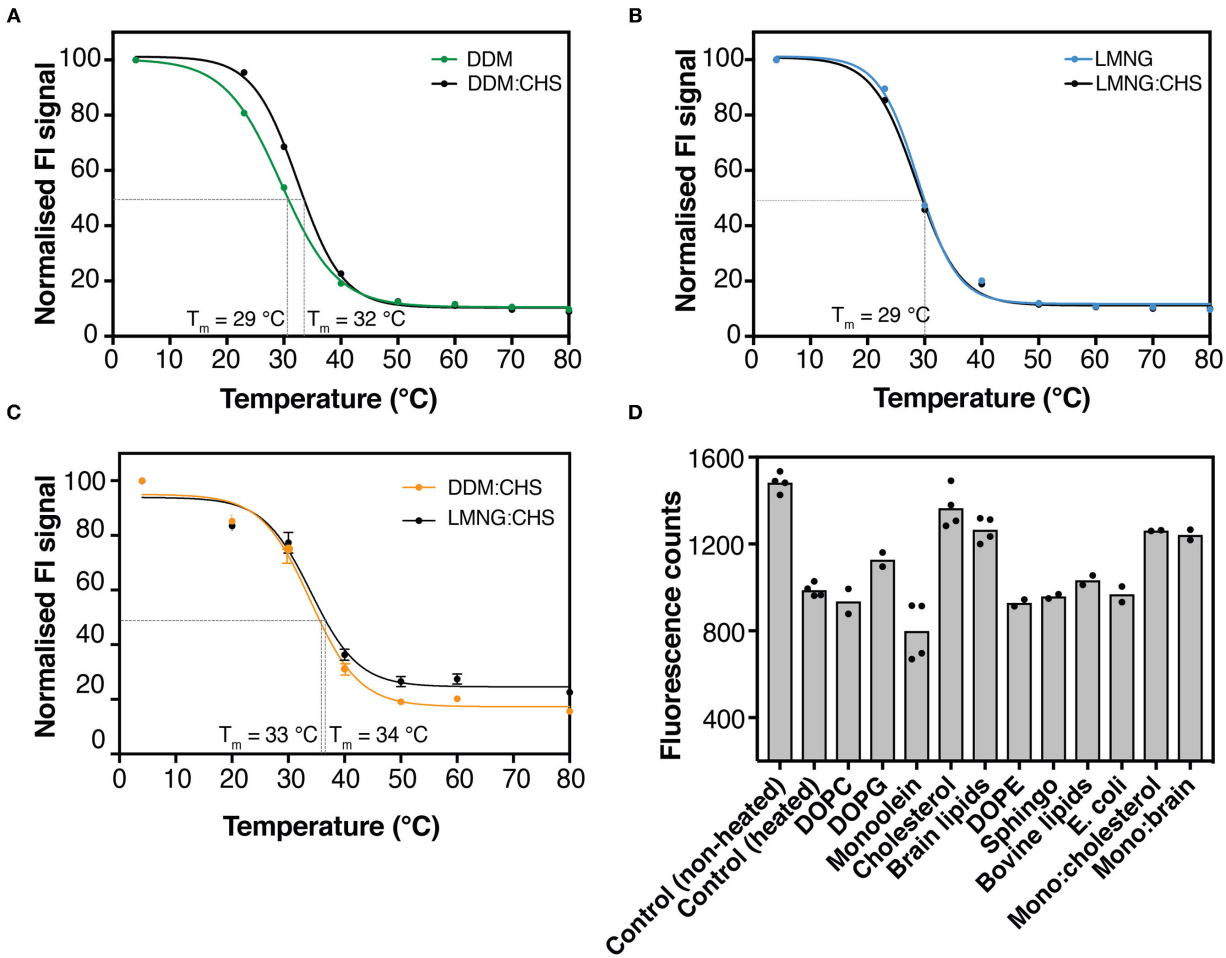
GFP-based thermal shift assay for hZnT1ΔC and hZnT1. **(A)** GFP-thermostability (GFP-TS) melting curves for crude-DDM solubilized hZnT1ΔC in the absence (green) and presence of CHS (black). **(B)** GFP-TS melting curves for crude-LMNG solubilized hZnT1ΔC in the absence (cyan) and presence of CHS (black). **(C)** GFP-TS melting curves for crude-DDM:CHS (orange) and LMNG:CHS (black) solubilized hZnT1. Curves were fitted to a sigmoidal dose-response curve as described in material and methods. **(D)** Effect of different lipids on the fluorescence counts of purified hZnT1ΔC before and after heat treatment; individual data points indicate technical replicates.

Obtaining folded protein is a key challenge when producing eukaryotic membrane proteins in unicellular organisms. *S. cerevisiae* cells have been used successfully for the production of active membrane proteins for both functional and structural characterization (Jidenko et al., 2005; Nomura et al., 2015; Schütz et al., 2016). This organism offers the simplicity of a unicellular system and lower costs in comparison to mammalian cell lines, and can provide some post-translation modifications (Vieira Gomes et al., 2018). However, *S. cerevisiae* membranes lack cholesterol, which can be essential for stability of eukaryotic membrane proteins (Singh, 2017). Several studies have demonstrated that cholesterol analogs, such as cholesteryl hemisuccinate (CHS), can stabilize some mammalian receptors for structural studies (Hanson et al., 2008; Jaakola et al., 2008; Shimamura et al., 2011; Xu et al., 2011). To evaluate the effect of CHS on the stability of hZnT1ΔC, DDM and LMNG solutions were supplemented with 0.2% (w/v) CHS. Our results, on the basis of GFP fluorescence levels, indicate that the addition of CHS improved detergent extraction efficiency. We also noted a slightly higher melting temperature (*T*_*m*_) for hZnT1ΔC in DDM-solubilized membranes supplemented with CHS (~3°C) relative to DDM (in the absence of CHS) or LMNG (in the absence or presence of CHS) solubilized membranes. Whereas, no significant difference in *T*_*m*_ were observed for hZnT1 in DDM and LMNG solutions supplemented with CHS (Figure 2).

We also investigated the effect of different lipids on the thermostability of hZnT1ΔC using GFP fluorescence (GFP-TS). We heated hZnT1ΔC to 5°C above its apparent *T*_*m*_ (~ 37°C) and compared the protein thermostability after supplementation with different lipids. As observed in Figure 2D, lipids such as DOPC, DOPE, sphingomyelin, bovine lipids and *E. coli* lipids had little or no effect on the thermostability of hZnT1ΔC, whereas DOPG, cholesterol and brain lipids improved the thermostability of the protein, where the measured fluorescence increased by 17, 47, and 30%, respectively. An interesting result was observed for monoolein, the typical host lipid for lipid cubic phase crystallography (LCP). Monoolein reduced the stability of the protein when compared to the non-heated control. However, addition of cholesterol or brain lipids (1:1) reversed this destabilizing effect of monoolein.

Taken together, the results indicate that the cholesterol analog CHS has a stabilizing effect on detergent solubilized hZnT1ΔC, and as such, was incorporated into subsequent protocols for purification and crystallization trials.

### Purified hZnT1ΔC Can Transport Zn^2+^ in Reconstituted Liposomes

ZnT1 has been described as a multifunctional protein, whose activities involve cell protection from zinc toxicity (Palmiter, 2004), inhibition of the L type calcium channels (LTCC) (Beharier et al., 2007; Shusterman et al., 2017), and activation of Ras-Raf-ERK signaling pathway (Bruinsma et al., 2002; Jirakulaporn and Muslin, 2004). More recently, Shusterman and co-workers demonstrated that ZnT1 from mammalian cells acts as a Zn^2+^/H^+^ exchanger protecting cells against zinc toxicity. This feature has been observed for zinc transporters (Chao and Fu, 2004; Ohana et al., 2009; Golan et al., 2019) and other members of the CDF family (Guffanti et al., 2002; Xu et al., 2019). Under this mechanism, the protein uses a proton drive force to transport divalent metals ions from the cytoplasm either to the outside of cells or into subcellular compartments (Chao and Fu, 2004).

**Figure 3:**
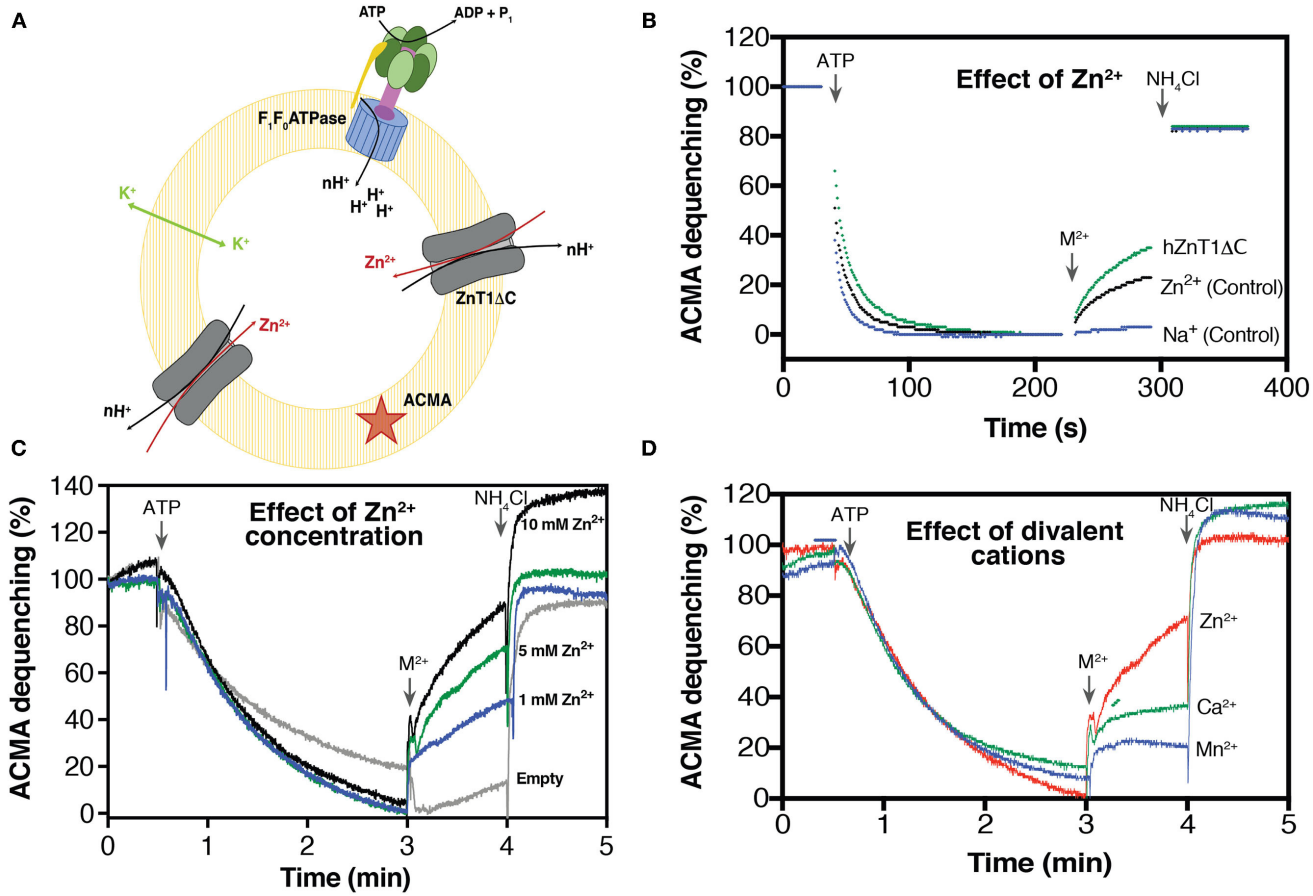
Substrate-induced H^+^ transporter activity of hZnT1ΔC. **(A)** Schematic representation of hZnT1ΔC and *E. coli* ATP synthase reconstituted in liposomes [L-α-Phosphatidylcholine: brain lipid (1:1)]. **(B)** Representative ACMA fluorescence traces for hZnT1ΔC antiporter activity in the presence of 10 mM ZnCl_2_ (green trace, *n* = 2). Black trace represents liposomes containing only ATP synthase (control, *n* = 3) in the presence of 10 mM ZnCl_2._ Blue trace represents liposomes containing only ATP synthase (control, *n* = 3) in the presence of Na^2+^. *n* corresponds to biological replicates. **(C)** Effect of Zn^2+^ concentrations on the activity of hZnT1ΔC. Empty liposomes correspond to liposome containing only ATP synthase. **(D)** Effect of divalent cations on hZnT1ΔC activity. Red line indicates Zn^2+^, blue line corresponds to Mn^2+^ whereas green lines indicates Ca^2+^. All metals were tested at concentration of 5 mM. A representative example of two biological replicates is shown.

To evaluate the antiporter mechanism of recombinant hZnT1, we reconstituted hZnT1ΔC and *E. coli* F_0_F_1_ ATP synthase into liposomes containing L-α-phosphatidylcholine and brain lipids (1:1 lipids ratio). In this experimental setup, ATP synthase acidifies the lumen of the liposomes by ATP-driven proton pumping, which is monitored by a decrease in the fluorescence of the signal molecule ACMA (Figure 3A). In hZnT1ΔC-containing proteoliposomes, the addition of Zn^2+^ ions will induce dequenching of ACMA fluorescence due to antiporter activity. To avoid the build-up of a membrane potential (Δψ), valinomycin and potassium are retained throughout the experiment, so that transport is primarily driven by the outwardly directed pH gradient. As shown in Figure 3B, an increase in fluorescence (ACMA dequenching) after addition of Zn^2+^ was detected for liposomes containing hZnT1ΔC. However, an increase in the signal was also observed for the control (ATP synthase with no hZnT1ΔC added). This behavior is likely due to the interaction of Zn^2+^ with the liposomes, since addition of Na^+^ (control) does not change the ACMA signal. Divalent cations can induce liposome fusion leading to leakage (Ellens et al., 1985), which may explain the increase in ACMA fluorescence. Indeed, Merriman and co-workers reported vesicle leakage after reconstitution of human ZnT8 in liposomes containing *E. coli* polar lipid extract (Merriman et al., 2016). Nevertheless, the induced response of the proteoliposomes containing hZnT1ΔC is measurably higher than the ATP synthase-only control, indicating that the recombinant hZnT1ΔC is functional. To determine an apparent *K*_*m*_ for Zn^2+^, the amount of ACMA dequenching over a range of various concentration of zinc was used and an apparent *K*_*m*_ of 1.42 mM was calculated (Figure 3C), similar to values observed for SoYiiP (Lopez-Redondo et al., 2020).

We also investigated the effect of Ca^2+^ and Mn^2+^ on the hZnT1 antiporter activity. Several studies have shown that some members of the CDF family recognize and transport other metals such as Cd^2+^ (Chao and Fu, 2004; Wei and Fu, 2005; Podar et al., 2012). In this assay, we found that hZnT1ΔC discriminates against Ca^2+^ and Mn^2+^ (Figure 3D), as low transport rates were observed in the presence of these two metals. This result agrees with reports of mammalian ZnT5 and ZnT8 activity (Hoch et al., 2012), suggesting that ZnT proteins have a high selectivity for Zn^2+^.

### hZnT1-CTD Can Be Produced in *E. coli* Cells

For expression of the C-terminal soluble domain (CTD) of hZnT1, the coding sequence for residues 342–507 was cloned into a pET vector encoding a C-terminal His-tag. As the gene sequence we used was originally optimized for yeast expression, and after rare codons analysis [Graphical Codon Usage Analyser (Fuhrmann et al., 2004)], we chose to express hZnT1-CTD in Rosetta-gami (DE3) cells containing the pRARE vector, which encodes tRNA genes for rare codons (Novy et al., 2001). After expression trials, we found the optimal conditions to be the use of auto-induction media at 30°C for 22 h.

Purification of hZnT1-CTD involved a multi buffer system using immobilized metal affinity chromatography (IMAC), SEC and anion exchange chromatography (MonoQ) (see Materials and Methods). As initial purification attempts in the presence of MES pH 6 buffer led to unstable protein at concentrations > than 5 mg/mL, thermo-shift assay was used to assess protein stability and to improve purification yield (see below).

### hZnT1-CTD Stability Increases at Low pH

To rapidly assess hZnT1-CTD stability in different conditions, we carried out a protein thermal shift assay using the Protein Thermal Shift^TM^ Dye Kit. In this assay, the Protein Thermal Shift dye binds to hydrophobic regions of proteins as they become solvent-exposed due to denaturation to give a fluorescent adduct.

**Figure 4:**
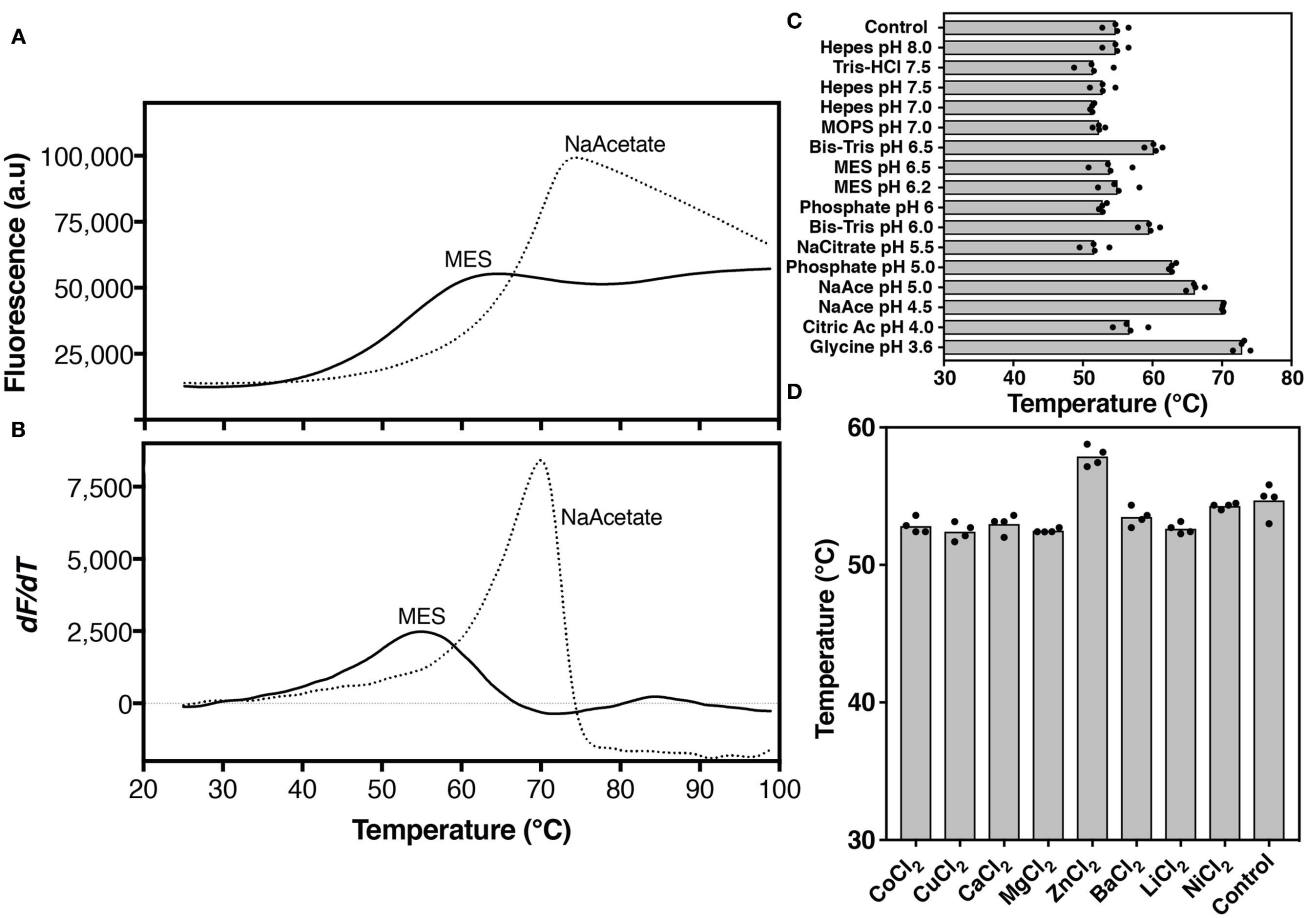
Thermal stability analysis of hZnT1-CTD. Protein unfolding was monitored by the fluorescence of Protein Thermal Shift dye. **(A)** Thermal denaturation profile, and **(B)** corresponding first derivative of hZnT1-CTD in MES buffer (solid line) and in the presence of 100 mM sodium acetate buffer pH 4.5 (dashed line). **(C)** Apparent *T*_*m*_ of hZnT1-CTD purified in MES buffer pH 6.0 (control) and under different buffers and pHs. **(D)** Apparent *T*_*m*_ of hZnT1-CTD purified in MES buffer pH 6.0 (control) in the presence of divalent cations. hZnT1-CTD at 1.0 mg/mL. Individual data points indicate technical replicates.

We observed during hZnT1-CTD purification that the protein tends to aggregate in MES buffer pH 6.0, at concentrations > 5 mg/mL. A low concentration of purified hZnT1-CTD (1 mg/mL) was incubated with buffers ranging from pH 3.0 to 10.0 (Supplementary Table 2). The results indicate that hZnT1-CTD is more stable at low pH, such as in buffers glycine-HCl pH 3.6 or sodium acetate pH 4.5 and 5.0 (Figure 4C). We chose to use sodium acetate pH 4.5 buffer for further evaluation of hZnT1-CTD stability.

hZnT1-CTD gave a typical sigmoidal profile, with an initial low fluorescence signal that transitions to a higher plateau as a temperature gradient induces the protein to unfold and expose its hydrophobic residues to the dye (Figure 4A). These measurements allow an apparent “melting” temperature (*T*_*m*_, a relative measure of protein stability) to be determined from the derivative of the profile (Figure 4B).

In MES buffer hZnT1-CTD exhibited an apparent T_m_ of 55°C. The apparent *T*_*m*_ of hZnT1-CTD increased significantly to 71°C in the presence of sodium acetate buffer pH 4.5 (Figure 4B). We also investigated the effect of divalent cations and additives on hZnT1-CTD stability. The results indicate that Zn^2+^, but not other divalent metals, slightly increases the apparent *T*_*m*_ from 55 to 58°C (Figure 4D). This result is not surprising, since previous studies have demonstrated that CTDs from zinc transporter orthologs bind Zn^2+^ ions (Cherezov et al., 2008). However, we expected a greater stabilization effect, since Zn^2+^ increased the apparent *T*_*m*_ of hZnT8 CTD by ~ 10°C (Parsons et al., 2018). The smaller increase in apparent *T*_*m*_ for hZnT1-CTD may be due to the presence of Zn^2+^ ions in the expression media (auto-induction media is supplemented with Zn^2+^). If the Zn^2+^ ions are carried through the purification steps they may occupy the stabilizing binding sites, as observed for SoYiiP (Lopez-Redondo et al., 2018). We note that the zinc binding site of the SoYiiP CTD has been described as the one with the highest affinity for zinc (Lu and Fu, 2007; Lu et al., 2009; Coudray et al., 2013).

Taken together, these results suggest that a low pH buffer and the presence of Zn^2+^ increase the stability of hZnT1-CTD. Therefore, the original purification protocol was modified to include a buffer exchange step, from Tris pH 7.5 into sodium acetate pH 4.5 buffer. This protocol led to a reproducibly stable protein solution with purity levels > 95%, which could be concentrated up to 12 mg/mL (Supplementary Figure 4).

### Crystallization of hZnT1 Variants

The generation of good quality crystals is often a rate-determining step in the determination of macromolecular structure of membrane proteins by X-ray crystallography (Suzuki et al., 2010; Batyuk et al., 2016). Several approaches have been implemented to increase crystallization success, including point mutations, truncations, addition of signal peptides and/or monoclonal antibodies, and insertion of water-soluble fusion proteins (Birch et al., 2018). We have shown that hZnT1ΔC is functional when fused with GFP, and we hypothesized that the presence of the GFP tag might assist hZnT1 crystallization, as has been shown for crystallization of some other small proteins (Suzuki et al., 2010).

Initial crystallization screens revealed crystals for both hZnT1 and hZnT1ΔC in several conditions (Supplementary Figure 5). Many of the hit conditions included divalent metals, mainly Ca^2+^ and Mg^2+^ but very few had Zn^2+^ present. This is possibly because zinc was carried through from expression through purification, and was already bound to the protein. Trials involving lipid cubic phase (LCP) crystallization were also performed and although we were able to identify a condition containing crystals, these disappeared over time and could not be reproduced (Supplementary Figure 6).

Further optimization of the crystals was carried out by varying protein and precipitant concentrations, pH, presence and absence of Zn^2+^, drop size and ratio, detergent type and by decreasing the temperature from 22 to 8°C. We found that several crystallization conditions were reproducible, but we could not obtain crystals larger than 50 μm, or measure diffraction beyond a resolution of 13 Å (Supplementary Figure 7).

The likely reasons for small crystals and poor diffraction are low purity and high conformational heterogeneity. A high level of purity is not essential for initial crystallization screening: it has been shown that 75% purity can be tolerated to obtain crystals using the vapor-diffusion technique (Kors et al., 2009). However, in that work, crystals obtained under those purity levels did not diffract beyond 10 Å (Kors et al., 2009). Although the fusion protein we crystallized was also found to be active, we estimate that the purity was below 80% for both hZnT1 and hZnT1ΔC and this could contribute to small crystal size and poor diffraction.

A high degree of conformational heterogeneity can inhibit crystallization or limit diffraction resolution. Our construct includes a 10-residue linker between the target and GFP fusion protein that could lead to conformational heterogeneity that inhibits crystal contact formation (Suzuki et al., 2010). Linkers of <5 residues have been used to crystallize soluble proteins fused to MBP or GFP (Center et al., 1998; Suzuki et al., 2010). We propose that a shorter linker and higher purity could yield larger and higher quality ZnT1 crystals.

We also attempted to crystallize hZnT1-CTD using the hanging drop vapor diffusion technique. However, no crystals were observed using this construct.

### hZnT1-CTD Forms Dimers With a V-Shaped Core

The CTD has been reported to play an important role in the mechanism of action of ZnT and other proteins belonging to the CDF family; however, its conformational changes upon Zn^2+^ binding are still unclear. Whilst some studies classify the CTD as a metal sensor that undergoes conformational changes upon zinc binding leading to changes on the transmembrane helices (TMH) (Lu et al., 2009), others suggest that the affinity between CTD and zinc is very high, such that zinc is always bound and conformational changes occur only in the TMD (Lopez-Redondo et al., 2018). The recently reported hZnT8 structure supports the latter contention as Zn^2+^ ions were bound to the CTD even though the samples were prepared in the absence of Zn^2+^. Furthermore, comparisons of the inward- and outward-facing conformations of ZnT8 strongly suggest that the CTD remains static during the transport cycle (Xue et al., 2020).

The hZnT8 structure confirms that its CTD is dimeric (Xue et al., 2020), as previously reported by Parsons and co-workers (Parsons et al., 2018), and as observed for bacterial CTD (Cherezov et al., 2008; Higuchi et al., 2009; Uebe et al., 2011; Zeytuni et al., 2014). However, a recent study showed that hZnT8-CTD can form a tetramer in solution (Ullah et al., 2020).

**Figure 5:**
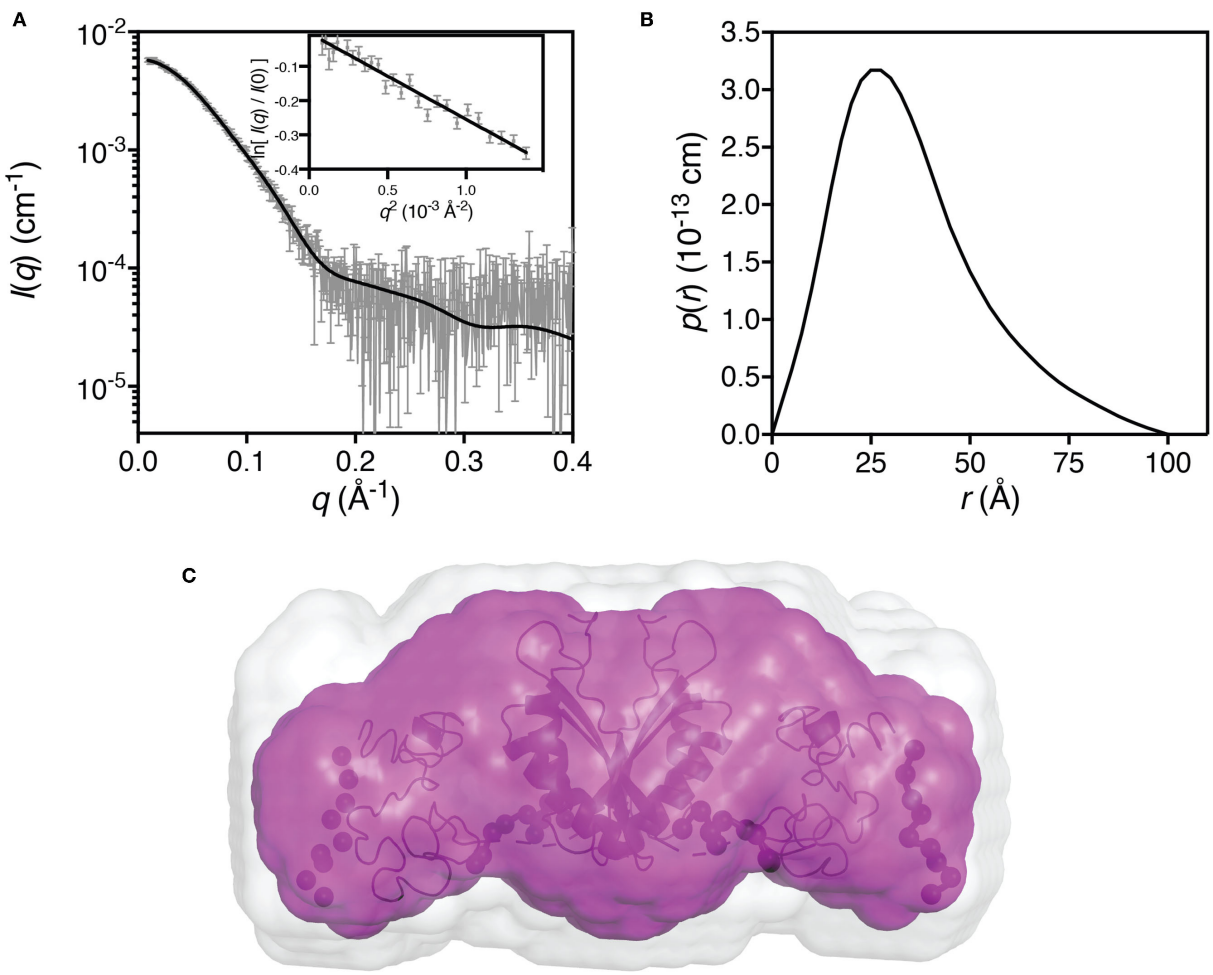
Small-angle X-ray scattering data for hZnT1-CTD. **(A)** Measured scattering data for hZnT1-CTD (gray). The scattering profile of the rigid-body model is shown as solid black lines overlaid on the scattering data (χ^2^ = 1.27; CorMap test (Franke et al., 2015), 475 points, *C* = 12, *P* = 0.108). **Insert:** Guinier plot for *qR*_*g*_ < 1.0 (*R*^2^ = 0.94); **(B)** The pair-distance distribution function, *p(r)*, derived from the scattering data are indicative of an elongated structure with a maximum dimension of ~100 Å; **(C)** Probable shape of the hZnT1-CTD dimer obtained from the filtered average of 16 dummy-atom models (magenta envelope): χ^2^ = 1.252 ± 0.002; NSD = 0.553 ± 0.026; Resolution = 26 ± 2 Å. Image in **C** was generated using PyMol (The PyMOL Molecular Graphics System, Schrödinger, LLC), where the light gray shape represents the total volume encompassed by the aligned dummy-atom models. The rigid-body model is shown as a black ribbon diagram aligned to the filtered model (flexible regions are represented by chains of black spheres).

We used small-angle X-ray scattering (Supplementary Table 3) to determine the oligomeric state and low-resolution solution structure of hZnT1-CTD (Figure 5). The estimated mass of the protein from the SAXS data is 41 kDa, indicating that the protein is present in solution as a dimer. Dummy atom modeling of the scattering data yields an elongated structure (Figure 5C, magenta envelope) whereas the rigid-body modeling against the scattering data yields a structure with a V-shaped core (Figure 5C, black ribbon representation), consistent with homologous proteins from prokaryotic organism. The homology structure was generated from the primary sequence using iTasser (Yang et al., 2014), and while good structure templates exist for residues 342–422, there are no structural templates for residues 422–507. As such, these residues were predicted by iTasser to be primarily coil (in a globular arrangement). Analysis of the sequence and small-angle scattering do not yield a consistent picture of the nature of this region of the protein. Sequence analysis shows that hZnT1-CTD has a longer tail than other homologs (Supplementary Figure 8) that is predicted to be disordered (Supplementary Figure 2). Rigid-body models optimized against the scattering data using the globular coil region as a “placeholder” yielded an excellent fit to the scattering data. While the predicted atomic structure of this region is unlikely to be representative of its actual structure, at low-resolution, the scattering data appears to be consistent with the notion that residues 422–507 are capable of forming a globular domain.

Previous studies suggest that the CTD of some CDF members adopts a more rigid and compact structure upon Zn^2+^ binding (Cherezov et al., 2008; Higuchi et al., 2009; Zeytuni et al., 2014; Ullah et al., 2020). However, the hZnT8-CTD structure shows no conformational differences between apo and Zn-bound CTD forms (Xue et al., 2020). This recent observation raises the question whether variations within the CTD caused by bound Zn^2+^ is species-dependent. Structural comparisons of our CTD model (large globular domain) with hZnT8-CTD gave an RMSD of 8 Å for 147 Cα atoms, with the main difference observed in helix-1, which led to a more compact structure in our model (Supplementary Figure 9). Whilst the RMSD is high, the overall model of hZnT1-CTD is in agreement with the hZnT8-CTD structure.

The hZnT8-CTD structure has two Zn^2+^ binding sites per monomer. However, the location and chemical environment of these are distinct from those in EcYiiP and SoYiiP. Whereas, Zn^2+^ ions have water molecules participating in the ion coordination in the YiiP structures, the hZnT8 map showed that the Zn^2+^ ions are in a classical tetrahedral geometry, with Zn^2+^ chelated by the HCH (His52-Cys53-His54) motif. This motif plays a role in Zn^2+^ transport, as its deletion leads to reduced Zn^2+^ transport rates (Xue et al., 2020). Although the HCH motif is conserved in ZnT8 homologs, it is not conserved in ZnT1 nor across the ZnT family; in fact, only ZnT2 and ZnT3 contain the motif. This raises questions about differences in the Zn^2+^ binding sites, the existence of other motifs that might be crucial for Zn^2+^ transport within the ZnT family, and whether the lack of the HCH motif would affect the two-state model of Zn^2+^ transport proposed (Xue et al., 2020).

Several studies have attempted to elucidate the structure-function relationship of the CTD of CDF members (Cherezov et al., 2008; Higuchi et al., 2009; Uebe et al., 2011; Zeytuni et al., 2014; Parsons et al., 2018; Ullah et al., 2020), showing that despite the high sequence divergence, all available CTD structures share a similar metallochaperone-like fold. However, the role of this domain in the transport mechanism of CDF members is still unclear. Xue and co-workers suggested from their structural studies of hZnT8 that the CTD along with TM3 and TM6 remain static between outward- and inward-facing states. Moreover, they pointed out that the CTD indirectly influences Zn^2+^ transport due to its interactions with the HCH motif, which restrict rearrangements in TMH1 leading to a wide cytosolic cavity in the inward conformation favoring Zn^2+^ binding (Xue et al., 2020). This knowledge contributes immensely to the field, though does not preclude distinct conformational changes occurring in other ZnTs and CDF members that lack the HCH motif, such as ZnT1.

## Conclusions

In the present study, we developed methods to reproducibly express and purify full-length human ZnT1, a C-terminal truncated variant (hZnT1ΔC), and its soluble CTD. Functional assays indicate that hZnT1ΔC has Zn^2+^/H^+^ antiporter activity. Crystallization of hZnT1 and hZnT1ΔC yielded crystals in several conditions; however, diffraction beyond 13 Å could not be achieved, suggesting that higher purity or a shorter linker may be required. Biochemical characterization of ZnT1-CTD indicates that this recombinant protein is stable at low pH (~4.5) and SAXS data support the formation of a dimer in solution that has a V-shaped core, similar to many other CTDs. These findings provide a basis for future structure-function studies of ZnT1 with potential for application to other ZnTs and CDFs. Follow-on experiments to increase our understanding of this class of proteins could include assessing: (i) the apparent Km of hZnT1 in comparison to hZnT1ΔC in liposome assays; (ii) the orientation of the protein after reconstitution in liposomes; (iii) the effect of different micelle sizes for hZnT1 and hZnT1ΔC as an alternative to investigate the role of the CTD in protein oligomerization and; iv) whether the combination of CHS and brain lipids can further increase protein stability.

## Data Availability Statement

The datasets presented in this study can be found in online repositories. The names of the repository/repositories and accession number(s) can be found in the article/Supplementary Material.

## Author Contributions

CC and RJ performed research, data analysis, and wrote the first draft. AW performed SAXS studies. HC conducted cloning and solubilization studies. JM and DD project conceptualization, project funding, and data analysis. All authors contributed to the draft of the manuscript and approved the final version.

## Conflict of Interest

The authors declare that the research was conducted in the absence of any commercial or financial relationships that could be construed as a potential conflict of interest.
