# Supplementary material for: Heterologous Expression and Biochemical Characterization of the Human Zinc Transporter 1 (ZnT1) and Its Soluble C-Terminal Domain
Source: Front Chem. 2021 Apr 30;9:667803. doi: 10.3389/fchem.2021.667803 (PMC8120272; doi:10.3389/fchem.2021.667803)
Supplement: Supplementary file 1 [file Data_Sheet_1.pdf]

frontiers

Supplementary Material

1 Supplementary Tables

Supplementary Table S1: list of primers

Primers	Sequence	Used for
hZnT1_Fwd	ACCCCGGATTCTAGACCTAGTGGATCCCCCATGGGTTGTTGG GGTAGAAACAGAGG	Cloning into pDDGFP vector
hZnT1_Rev	AAATTGACCTTGAAAATATAAATTTTCCCCCAAAGAG GATTCAGGTTGC	
hZnT1_Rev_t2	AAATTGACCTTGAAAATATAAATTTTCCCCCTTCAAAGCACATTG AGTTCTACAAGCCAATTCACATGGTTGAATGGTAGTGGCATG	For hZnT1ΔC truncation
hZnT1-CTD-NdeI-Fwd	GGAATTCCATATGATTTTGTTGCAAACCGTTCC	Cloning into pET24a vector
hZnT1-CTD-XhoI-Rev	CCGCTCGAGCAAAGAGGATTCAGGTTGCT	

Supplementary Table S2: Buffers, metals and chemicals used for thermostability assay.

	1	2	3	4	5	6	7	8	9	10	11	12
**A**	100mM Glycine pH 3.0	100mM Citric Acid pH 3.2	100mM Glycine pH 3.6	100mM Citric Acid pH 4.0	100mM Sodium Acetate pH 4.5	100mM Sodium Acetate pH 5.0	100mM Phosphate pH 5.0	100mM Sodium Citrate pH 5.5	100mM Bis Tris pH 6.0	100mM Phosphate pH 6.0	100mM MES pH 6.2	100mM MES pH 6.5
**B**	100mM Bis Tris Propane pH 4.5	100mM MOPS pH 7.0	100mM Phosphate pH 7.0	100mM HEPES pH 7.0	100mM HEPES pH 7.5	100mM Tris HCl pH 7.5	100mM HEPES pH 8.0	100mM Tris HCl pH 8.0	100mM Bicine pH 8.5	100mM Tris HCl pH 8.5	100mM CHES pH 9.0	100mM CHES pH 9.5
**C**	100mM CAPS pH 10	80mM NaCl	400mM NaCl	1M NaCl	20% Glycerol	40% Glycerol	200mM Glucose	200mM Sucrose	100µM $CoCl_2$	100µM $CuCl_2$	100µM $CaCl_2$	100µM $MgCl_2$
**D**	100µM $ZnCl_2$	100µM $BaCl_2$	100µM $LiCl_2$	100µM $NiCl_2$	50mM $(NH_4)_2SO_4$	500mM $(NH_4)_2SO_4$	Water Minus Protein	Water Plus Protein	Protein Buffer Minus Protein	Protein Buffer Plus Protein	Lysozyme Minus Protein	Lysozyme Plus Protein

Supplementary Table S3: SAXS data collection and analysis details

**Data Collection Parameters**	**hZnT1-CTD**
Instrument	SAXS-WAXS (Australian Synchrotron)
Beam geometry	250 µm (h) × 450 µm (v)
Wavelength (Å)	1.078
Sample to detector distance (m)	2.791
q-range (Å^{-1})	0.005 – 0.50
Exposure time (s) *	30 (30 × 1 s exposures)
Configuation	In-line SEC-SAXS (S200 5/150 GL) with sheath flow
Injection concentration (mg/mL)	1.5
Injection volume (µL)	60
Flow rate (mL/min)	0.45
Temperature (K)	283
Absolute intensity calibration	Water
**Sample details**	
Extinction coefficient (A_{280}, 0.1% *w/v*)	0.383
Partial specific volume (cm^{3}g^{-1})	0.739
Contrast, $\Delta\rho$ (10^{10} cm^{-2})	2.84
Molecular mass [from sequence] (kDa)	18.3
Protein concentration (mg/mL) §	-
**Structural parameters**	
$I(0)$ (cm^{-1}) [from Guinier]	0.00594 ± 0.00005
R_g (Å) [from Guinier]	27.5 ± 0.6
$I(0)$ (cm^{-1}) [from $p(r)$]	0.00594 ± 0.00003
R_g (Å) [from $p(r)$]	27.9 ± 0.2
D_{max} (Å)	100 ± 3
Porod volume (Å^3)	50000 ± 2500
**Molecular mass determination**	
Molecular mass [from Porod] (kDa)	41 ± 2
**Software employed**	
Primary data reduction	*Scatterbrain* (v 2.71)
Data processing	*PRIMUS* (v 3.2) and *GNOM* (v 4.6)
Ab initio modelling	*DAMMIN* (v 5.3)
Validation and averaging	*DAMAVER* (v 2.8.0)
Rigid body modelling	*CORAL* (v 1.1)
Three-dimensional graphics	*PyMOL*

*The *Rg* across the 30 averaged frames shows no systematic trend, hence, it is deemed that there are no significant interparticle interactions present in the data.

§The actual protein concentration is unknown, but assuming a protein mass of 40kDa, from $I(0)$, the average protein concentration is estimated to be ~0.2 mg/mL.

2 Supplementary Figures

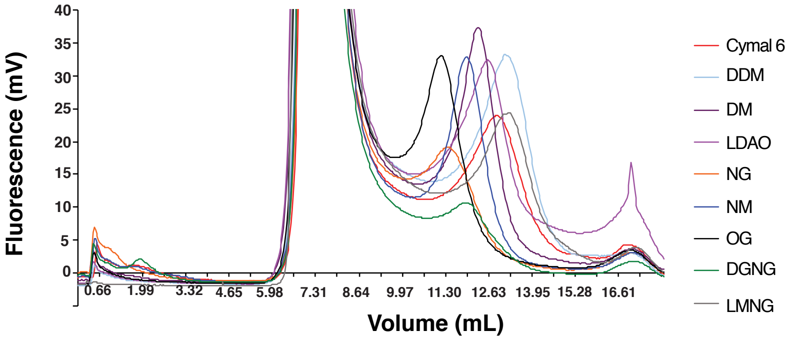

Supplementary Figure 1. Detergent screening for hZnT1 analysis. Protein was solubilized in each detergent and analyzed by FSEC as described in material and methods.

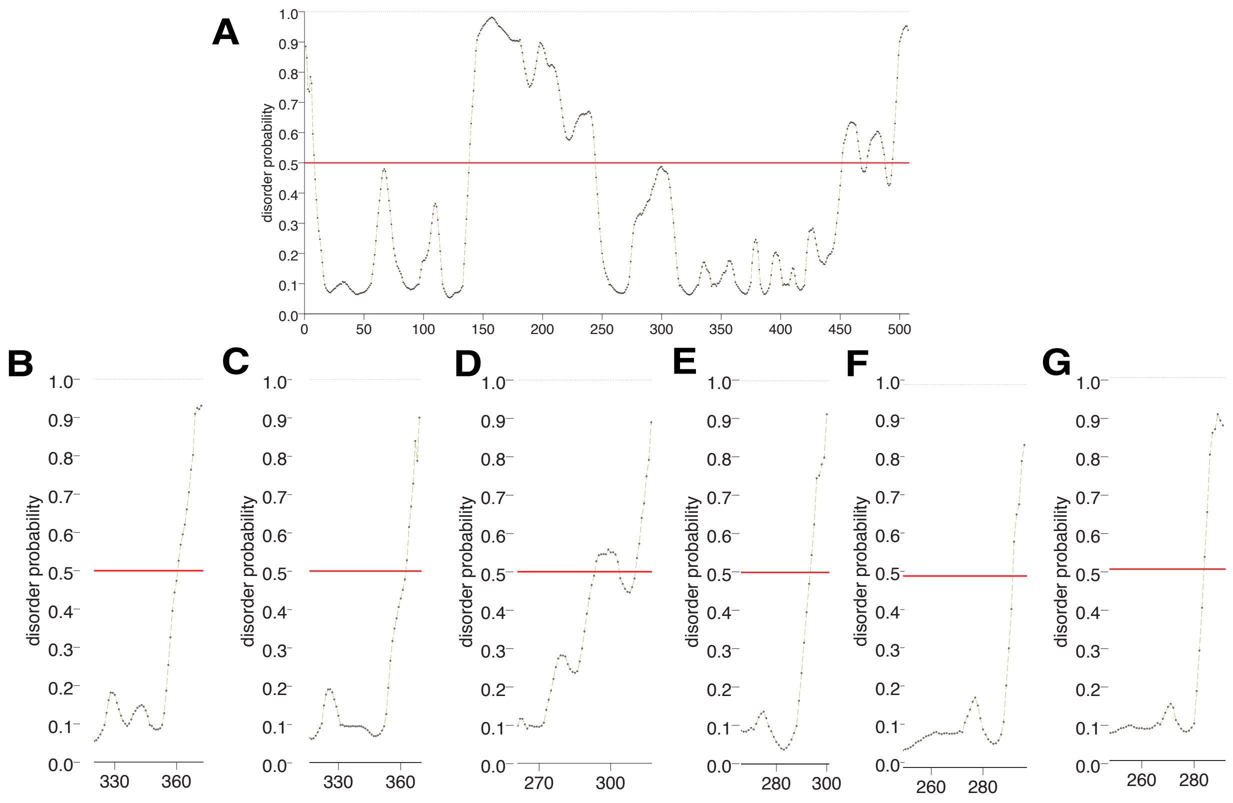

Supplementary Figure 2. Disorder prediction of the full-length hZnT1 and the C-terminal domains of other CDF members. Disorder regions were predicted using Protein Disorder Prediction System (PrDOS) server (Ishida and Kinoshita, 2007). The red line indicates 95% confidence of disorder. **(A)** ZnT1 from *Homo sapiens*; **(B)** ZnT2-CTD from *Homo sapiens*; **(C)** ZnT8-CTD from *Homo sapiens;* **(D)** Magnetosome protein (MamM-CTD) from *Magnetospirillum gryphiswaldense*; **(E)** YiiP-CTD from *Escherichia coli*; **(F)** YiiP-CTD from *Shewanella oneidensis*; **(G)** CzrB-CTD from *Thermus thermophilus.*

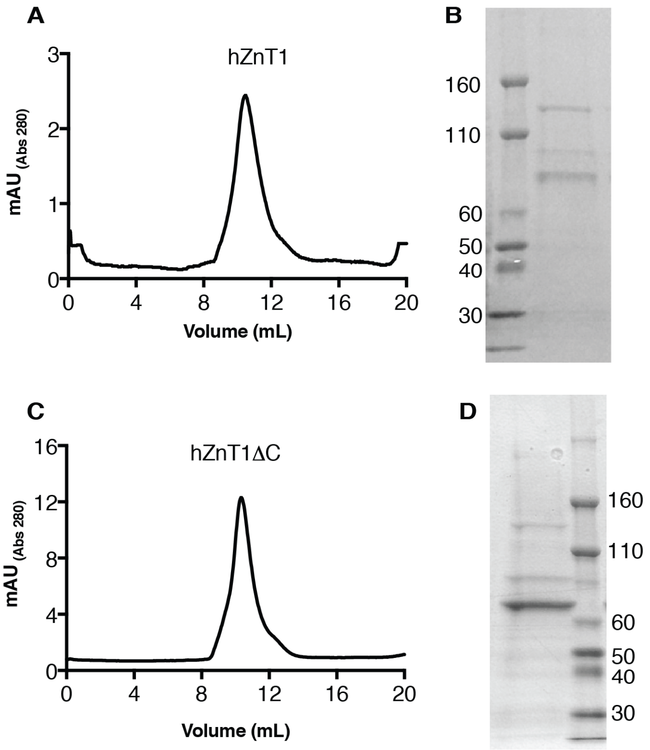

Supplementary Figure 3. Size exclusion chromatography of purified hZnT1 and hZnT1ΔC. **(A)** SEC profile of hZnT1 after a two step purification. Protein was loaded into a Superdex 200 10/300 and shows a monodisperse peak at 10 mL. **(B)** Coomassie-stained gel showing molecular weight markers (left lane) and the hZnT1 sample (right lane) (MW: 87.8 kDa). **(C)** SEC profile of hZnT1ΔC after a two step purification. Protein was loaded onto a Superdex 200 10/300. **(D)** Coomassie-stained gel showing molecular weight markers (left lane) and the hZnT1ΔC sample (right lane) (MW: 79.7 kDa).

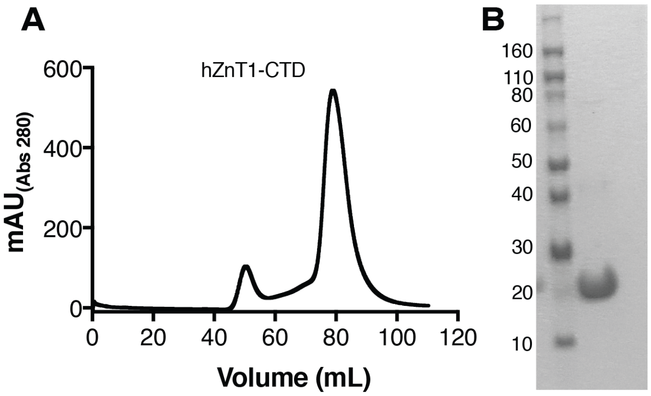

Supplementary Figure 4. Size exclusion chromatography of IMAC purified hZnT1-CTD. **(A)** SEC profile of hZnT1-CTD after IMAC purification. Protein was loaded onto a Superdex 200 16/60 column and shows a monodisperse peak at 78 mL. Buffer: 25 mM sodium acetate pH 4.5, 100 mM NaCl, 0.5 mM TCEP; **(B)** Coomassie-stained gel under reducing condition showing molecular weight markers (left lane) and the purified hZnT1-CTD (right lane) (MW:19.5 kDa) after ion exchange chromatography.

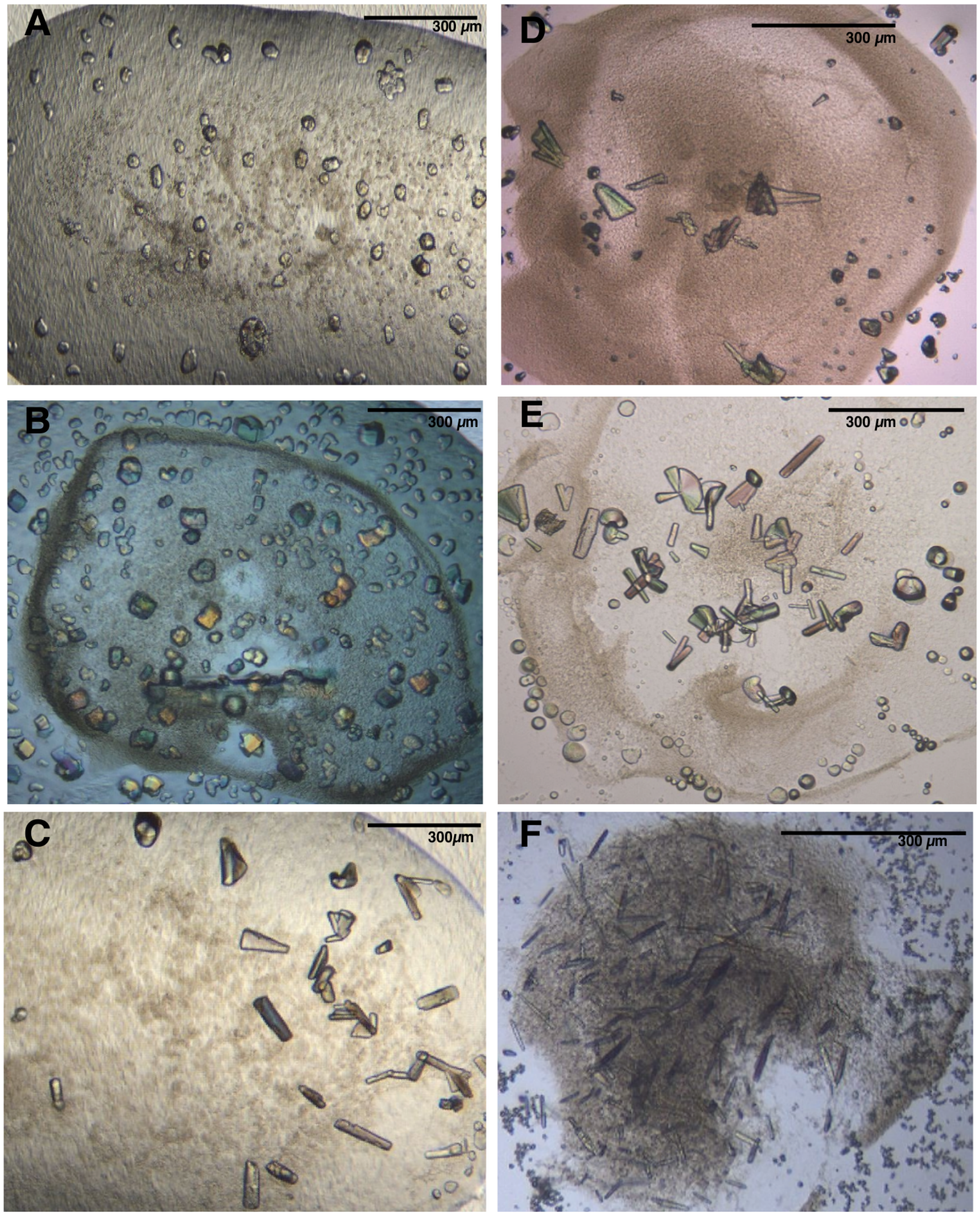

Supplementary Figure 5. Outcome of crystallization trials of ZnT1 and hZnT1ΔC**.** Proteins were purified in buffer containing 20 μM of $ZnCl_2$. **(A)** hZnT1 (10 mg/mL). Crystallization condition: 32% (v/v) PEG 400, 0.05 M $Mg(CH_3COO)_2$, 0.1 M Glycine pH 9.5. First crystals appeared after day 21; **(B)** hZnT1 (10 mg/mL). Crystallization condition: 32% (v/v) MPEG550, 0.1 M Tris-HCl pH 8.5. First crystals appeared after day 21 **(C)** hZnT1 (10 mg/mL). Crystallization condition: 30% (v/v) PEG 200, 0.1 M MES pH 6.0, 0.1 M NaCl, 0.1 M $CaCl_2$. Crystals appeared after day 80. **(D)** hZnT1ΔC (11 mg/mL). Crystallization condition: 29% (v/v) PEG 400, 0.05 M $Ca(C_2H_3O_2)_2$ pH 5.0, 0.2 M NaCl. First crystals appeared after day 21. **(E)** hZnT1ΔC (6 mg/mL). Crystallization condition: 28% (v/v) PEG 400, 0.1 M MES pH 6.5, 0.03 M $MgCl_2$. First crystal appeared after day 57. **(F)** hZnT1ΔC (11.5 mg/mL). Crystallization condition: 26% (v/v) PEG 400, 0.1 M MES pH 6.7, 0.05 M $Mg(CH_3COO)_2$. First crystals appeared after day 13.

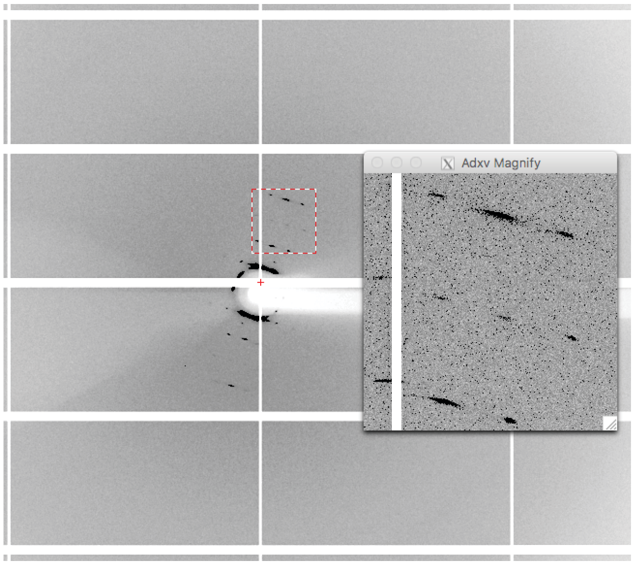

Supplementary Figure 6. X-ray diffraction from a hZnT1 crystal. X-ray diffraction from a crystal harvested from Suppl Figure 5C crystallization drop. Diffraction was measured on a ADSC Quantum 315r detector at the MX2 beamline, Australian Synchrotron.

```
HsZnT1  288 PDPCKAFVEIINSTHASVYEAGPCWVLYLDPTLCVVMVCILLYTTYPLLKESALILLQTVPKQIDIRNLIKELRN--VEGVEEVHELH 373
HsZnT2  245 -----------------------PEYKYVDPICTFVFSILVLGTTLTILRDVILVLMEGTPKGVDFTAVRDLLLS--VEGVEALHSLH 307
HsZnT8  242 -----------------------PEYKIADPICTFIFSILVLASTITILKDFSILLMEGVPKSLNYSGVKELILA--VDGVLSVHSLH 304
MgMamM  177 -----------------------NMP-WIDPAVALWETIDLLLLGKVVFMDAYRGLMDHTAGEAVQNRIVEAAER--VPGVRGVIHLR 238
TtCzrB  168 -----------------------GLW-VLDPLLALAVAGQILFLGYRIVRESVGGLMDEGLPPEEVERIRAFLQERIRGRALEVHDLK 231
EcYiiP  174 -----------------------GWH-RADALFALGIGIYILYSALRMGYEAVQSLLDRALPDEERQEIIDIVTS--WPGVSGAHDLR 235
SoYiiP  176 -----------------------GWV-WADGLFAVLIACYIGQQAFDLGYRSIQALLDRELDEDTRQRIKLIAKE--DPRVLGLHDLR 237

HsZnT1  374 VWQLAGSRIIATAHIKCEDPT---SYMEVAKTIKDVFHNH--GIHATTIQPEFASVGSKSSVVPCELACRTQCALKQCCGTLPQAPSG 456
HsZnT2  308 IWALTVAQPVLSVHIAIAQNT---DAQAVLKTASSRLQGKF-HFHTVTIQIEDYS-EDMKDCQACQGPSD------------------ 372
HsZnT8  305 IWSLTMNQVILSAHVATAASR---DSQVVRREIAKALSKSF-TMHSLTIQMESPVDQDP-DCLFCEDPCD------------------ 369
MgMamM  239 A-RYVGQDIWADMIIGVDPENTVEQAHEICEAVQAAVCGKIRRIESLHVSAEAREIGDTTKPSFSDQPLSFDEVMLSKVDN------- 318
TtCzrB  232 T-RRAGPRSFLEFHLVVRGDTPVEEAHRLCDELERALAQAF-PGLQATIH------VEPEGERKRTNP-------------------- 291
EcYiiP  236 T-RQSGPTRFIQIHLEMEDSLPLVQAHMVADQVEQAILRRF-PGSDVIIH------QDPCSVVPREGKRSMLS--------------- 300
SoYiiP  238 T-RQAGKTVFIQFHLELDGNLSLNEAHSITDTTGLRVKAAF-EDAEVIIH------QDPVQVEPTTQ--------------------- 296

HsZnT1  457 KDAEKTPAVSISCLELSNNLEKKPRRTKAENIPAVVIEIKNMPNKQPESSL 507
HsZnT2      ---------------------------------------------------
HsZnT8      ---------------------------------------------------
MgMamM      ---------------------------------------------------
TtCzrB      ---------------------------------------------------
EcYiiP      ---------------------------------------------------
SoYiiP      ---------------------------------------------------
```

Supplementary Figure 7. Sequence alignment of the C-terminal domains. The CTD of human zinc transporters and bacterial CDF members are shown. Alignment was prepared using MUSCLE (Edgar, 2004) and Jalview (Waterhouse et al., 2009). The black arrow indicates the beginning of the soluble C-terminal domain. Accession numbers are as follows: *Homo sapiens* ZnT1 (HsZnT1), Q9Y6M5; *Homo sapies* ZnT2 (HsZnT2), Q9BRI3-2; *Homo sapiens* ZnT8 (HsZnT8), Q8IWU4; *Magnetospirillum gryphiswaldense* MamM (MgMamM), V6F235; *Thermus thermophilis* CzrB (TtCzrB), Q8VLX7; *Escherichia coli* YiiP (EcYiiP), P69380; *Shewanella oneidensis* YiiP (SoYiiP), Q8E919.

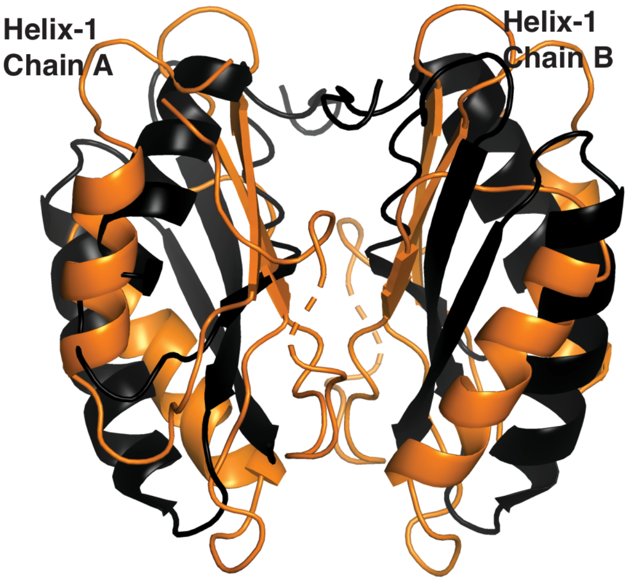

Supplementary Figure 8. Structural comparison of hZnT1-CTD model and hZnT8-CTD structure. Superposition of hZnT1-CTD SAXS model (black) with hZnT8-CTD dimer (orange) (PDB code: 6XPE; RMSD of 7.87 Å for 147 Cα atoms).

Supplementary Note 1. The codon optimized gene sequence used for expression of hZnT1

```
ATGGGTTGTTGGGGTAGAAACAGAGGTAGATTATTGTGTATGTTGGCCTTGACCTTCATGTTCATGGTTTTGGA
AGTTGTTGTTTCCAGAGTCACTTCTTCTTTGGCTATGTTGTCTGATTCCTTCCACATGTTGTCAGATGTTTTGG
CTTTGGTTGTTGCATTGGTTGCTGAAAGATTTGCTAGAAGAACTCATGCTACTCAAAAGAACACTTTCGGTTGG
ATTAGAGCTGAAGTTATGGGTGCTTTGGTTAACGCTATTTTCTTGACTGGTTTGTGCTTCGCCATTTTGTTGGA
AGCTATTGAAAGATTCATCGAACCACACGAAATGCAACAACCTTTAGTTGTTTTGGGTGTTGGTGTAGCTGGTT
TGTTGGTTAATGTTTTAGGTTTGTGTTTGTTCCACCACCACTCTGGTTTTTCTCAAGATTCTGGTCATGGTCAT
TCTCATGGTGGTCACGGTCATGGTCACGGTTTGCCAAAAGGTCCAAGAGTTAAGTCTACTAGACCAGGTTCTTC
CGATATTAACGTTGCTCCAGGTGAACAAGGTCCAGATCAAGAAGAAACTAATACCTTGGTTGCTAACACCTCTA
ACTCTAACGGTTTGAAATTGGATCCAGCCGATCCAGAAAATCCAAGATCTGGTGATACTGTTGAAGTTCAAGTC
AACGGTAACTTGGTTAGAGAACCAGATCATATGGAATTGGAAGAAGATAGAGCTGGTCAATTGAATATGAGAGG
TGTTTTCTTGCATGTCTTGGGTGATGCTTTGGGTTCTGTTATCGTTGTTGTTAACGCTTTGGTTTTCTACTTCT
CTTGGAAGGGTTGTTCTGAAGGTGATTTTTGTGTTAACCCATGTTTCCCAGATCCATGTAAGGCTTTCGTTGAA
ATTATCCAATCTACCCATGCCTCTGTTTATGAAGCTGGTCCATGTTGGGTCTTGTACTTAGATCCAACTTTGTG
TGTTGTTATGGTCTGCATCTTGTTGTACACTACTTACCCTTTGTTGAAAGAATCCGCCTTGATTTTGTTGCAAA
CCGTTCCAAAGCAAATCGACATCAGAAACTTGATCAAAGAATTGAGAAACGTCGAAGGTGTTGAAGAAGTTCAC
GAATTGCACGTTTGGCAATTGGCTGGTTCTAGAATTATTGCTACCGCTCATATTAAGTGCGAAGATCCAACATC
CTATATGGAAGTTGCTAAGACCATCAAGGATGTTTTCCATAACCATGGTATTCATGCCACTACCATTCAACCAG
AATTTGCTTCTGTTGGTTCCAAGTCATCTGTTGTTCCATGTGAATTGGCTTGTAGAACTCAATGTGCTTTGAAG
CAATGTTGTGGTACTTTGCCACAAGCTCCATCTGGTAAAGATGCTGAAAAAACTCCAGCCGTTTCTATCTCTTG
TTTGGAATTGTCTAACAACTTGGAAAAGAAGCCAAGAAGAACAAAGGCCGAAAACATTCCAGCTGTTGTTATCG
AAATCAAGAACATGCCAAACAAGCAACCTGAATCCTCTTTG
```

References

Edgar, R. C. (2004). MUSCLE: multiple sequence alignment with high accuracy and high throughput. *Nucleic Acids Res.* 32, 1792–7. doi:10.1093/nar/gkh340.

Ishida, T., and Kinoshita, K. (2007). PrDOS: prediction of disordered protein regions from amino acid sequence. *Nucleic Acids Res.* 35. doi:10.1093/nar/gkm363.

Waterhouse, A. M., Procter, J. B., Martin, D. M. A., Clamp, M., and Barton, G. J. (2009). Jalview Version 2--a multiple sequence alignment editor and analysis workbench. *Bioinformatics* 25, 1189–91. doi:10.1093/bioinformatics/btp033.
